# Assessment of Electrocardiogram (ECG) Interpretation Proficiency Among Paramedics in a Tertiary Care Teaching Hospital

**DOI:** 10.7759/cureus.95881

**Published:** 2025-11-01

**Authors:** Chinnam Vishnupriya, Nitheesha Thomas, Gireesh Kumar, Sreekrishnan Trikkur, Annet Maria Thomas, Sai Suprathika Ponnaboina, Devika Saju

**Affiliations:** 1 Emergency Medicine, Amrita Institute of Medical Sciences, Kochi, IND; 2 Medical Administration, Ganga Hospital, Coimbatore, IND; 3 Dietetics, Hyderabad Rheumatology Centre (HRC), Hyderabad, IND; 4 Biostatistics, Amrita Institute of Medical Sciences, Kochi, IND

**Keywords:** cardiac emergencies, clinical training, competency, diagnostic accuracy, ecg interpretation, emergency medicine, healthcare education, lifesaving intervention, medical simulation, paramedic education

## Abstract

Background

Electrocardiogram (ECG) interpretation is a critical clinical skill essential for the rapid diagnosis of potentially life-threatening cardiac conditions, such as arrhythmias and myocardial infarctions. Accurate interpretation plays a pivotal role in clinical decision-making, particularly in emergency settings. Paramedics often acquire practical ECG experience during clinical rotations under the guidance of experienced clinicians. Familiarity with standardized guidelines, such as those from the American Heart Association, is fundamental for ensuring accurate and consistent ECG assessments.

Aim

This study aimed to evaluate the proficiency in ECG interpretation among paramedics at various training and professional levels in a tertiary care teaching hospital, with the goal of improving recognition and resuscitation outcomes in in-hospital cardiac arrests.

Materials and methods

A cross-sectional observational study was conducted using a validated, self-administered online questionnaire distributed via Google Forms. A total of 90 participants, including BSc Emergency Medical Technology (EMT) and BSc Intensive Care Technology (ICT) students, interns, emergency medical technician (EMT) staff, and emergency room (ER) nursing staff at Amrita Institute of Medical Sciences (AIMS), were surveyed. The questionnaire comprised 15 structured questions assessing ECG interpretation skills. Participants were categorized based on their scores: 0-5 (poor knowledge), 6-10 (average knowledge), and 11-15 (good knowledge). Statistical analysis was conducted using SPSS 20.0, with Kruskal-Wallis and Spearman's correlations applied.

Results

Among the 90 participants, 66 (73.3%) demonstrated good knowledge, while 24 (26.7%) showed average knowledge; no participant fell into the poor category. The mean total score was 11.86±2.33. Third-year students (mean: 13.21±1.87), interns (12.26±1.59), and staff (12.32±1.92) outperformed second-year students (9.57±2.34). The Kruskal-Wallis test revealed significant differences in the scores across training levels (χ²=26.024, *p*<0.001). While overall proficiency was high, second-year students and ER nurses scored comparatively lower on specific advanced ECG questions.

Conclusion

The findings indicate a generally high level of ECG interpretation knowledge among paramedics, especially among advanced-level students. However, there is a clear need for targeted educational interventions and structured training programs, particularly for early-stage students and ER nursing personnel, to strengthen foundational skills in ECG interpretation. The interpretation skills acquired by the personnel have helped them in recognizing ECG rhythms that result in cardiac arrest, which pave the way for early acknowledgment and management.

## Introduction

Electrocardiography (ECG) is a vital diagnostic tool in emergency and pre-hospital care, enabling rapid assessment of a patient's heart condition. For paramedics and emergency responders, the ability to appropriately interpret ECGs is crucial for the prompt detection and management of life-threatening cardiac disorders, including arrhythmias, myocardial infarctions (MI), and conduction abnormalities, e.g., bundle branch blocks (BBB) that directly influence resuscitation and triage decisions [1].

The American Heart Association emphasizes the importance of prompt rhythm identification and standardized interpretation as a core component of high-quality resuscitation [2]. Despite its pivotal significance, numerous trainees and healthcare professionals exhibit considerable variability in the accuracy of ECG interpretation. Recent systematic reviews and meta-analyses reveal that the average test accuracy of physicians and trainees often lags behind optimal standards, but structured, competency-based training significantly enhances interpretative performance [3,4].

These evaluations indicate that although fundamental rhythm detection may be taught well, the identification of nuanced ischemia alterations and intricate arrhythmias remains unreliable without specialized educational initiatives.

Accurate ECG interpretation necessitates the reliable identification of essential rhythms, ischemia alterations, and conduction irregularities to effectively inform patient triage and urgent decision-making. Previous research indicates that high-stakes clinical judgments often necessitate approximately 80% accuracy in crucial patterns to be deemed safe [5].

In the high-pressure context of emergency medical services (EMS), paramedics frequently serve as the initial evaluators of patients presenting with chest discomfort, palpitations, or syncope. Precise ECG interpretation accelerates essential actions and assesses the urgency of hospital transfer [6]. Nonetheless, significant diversity remains, shaped by training background, exposure, and confidence [7]. Although numerous paramedics consistently recognize prevalent abnormalities such as atrial fibrillation or ventricular tachycardia, nuanced findings like non-ST elevation myocardial infarctions (NSTEMIs) or BBB provide greater challenges [8].

Recent systematic evaluations indicate that periodic refresher training, simulation-based learning, and customized curriculum enhance ECG interpretation skills [3,9]. Nevertheless, research from Ethiopia and Saudi Arabia indicates persistent inadequacies in ECG knowledge among emergency nurses and paramedics. Comparable data from India is scarce, especially in tertiary teaching hospitals [6,10].

Moreover, attaining approximately 80% accuracy in identifying essential ECG patterns is widely regarded as a clinical standard for safe and successful interpretation, highlighting the necessity for focused competency-based education. Incorporating diverse professional groups such as paramedic students, interns, emergency medical technician (EMT) staff, and emergency room nursing personnel facilitates a thorough assessment of proficiency across different levels of clinical training and exposure, thereby guiding customized educational strategies to enhance outcomes in emergency cardiac care [5,11].

Considering these deficiencies, assessing ECG competencies among various professional groups such as paramedic students, interns, emergency medical technician (EMT) staff, and emergency room nursing personnel provides significant insights. Analyzing groups at various training phases reveals strengths in advanced participants and limitations in earlier trainees or non-physician staff, therefore guiding the development of targeted interventions.

Accordingly, this study seeks to assess the proficiency in ECG interpretation among paramedic students, interns, EMT staff, and emergency room nursing personnel in a tertiary care teaching hospital. It specifically analyzes competence disparities across training stages, positing that advanced students and staff surpass early-stage learners. The results will guide the development of customized educational interventions targeting identified deficiencies in ECG proficiency to enhance emergency cardiac care outcomes.

## Materials and methods

Study design and setting

This study employed a cross-sectional survey to assess ECG interpretation proficiency among paramedics and nursing personnel at Amrita Institute of Medical Sciences and Research Centre (AIMS), Kochi - a tertiary care teaching hospital in South India. Data collection was conducted over a three-month period (June-August 2024).

Study population and sample size

A preliminary pilot investigation was conducted with 10 volunteers to assess the average ECG interpretation score (13.4±2.36). Using this data, the minimum required sample size was estimated as 48, assuming a medium effect size (Cohen’s d=0.6), a 5% level of significance (α=0.05), and 80% statistical power (1-β=0.80) with a 95% confidence interval.

All eligible participants from the Emergency Department were invited to participate. Of the 90 individuals approached, all consented and completed the questionnaire, resulting in a final sample size of 90, which exceeded the minimum requirement and further strengthened the statistical power of the analysis.

Eligibility criteria

The inclusion criteria were the following: (i) undergraduate BSc students in Emergency Medical Technology (EMT) and Intensive Care Technology (ICT); (ii) EMT/ICT interns and EMT staff working in the Emergency Department; (iii) emergency room nursing personnel actively involved in patient care; and (iv) participants who provided written informed consent.

The exclusion criteria included individuals not directly involved in emergency care activities, incomplete or duplicate survey responses, or those who declined or withdrew consent.

Ethical considerations

The study was approved by the Institutional Ethical Committee (IEC) of Amrita Institute of Medical Sciences (ID: ECASM-AIMS-2025-332, dated July 31, 2025). All the qualified candidates from the emergency department were invited to participate on their own accord. Each of the 90 eligible candidates provided informed consent and completed the questionnaire that was provided. Careful preservation of confidentiality and anonymity for participants was maintained throughout the study.

Data collection tools and validation

A structured self-administered online questionnaire was created in accordance with the 2020 American Heart Association (AHA) guidelines for ECG interpretation and Advanced Cardiovascular Life Support (ACLS) standards.

The questionnaire comprised 15 multiple-choice questions covering five ECG domains: (1) recognition of rhythms (five items): normal sinus rhythm, atrial fibrillation, ventricular tachycardia, ventricular fibrillation, and asystole; (2) ST-T segment abnormalities (three items): ST elevation, ST depression, and mild T-wave inversion; (3) conduction blocks (two items): right bundle branch block (RBBB) and left bundle branch block (LBBB); (4) hypertrophy patterns (two items): left ventricular hypertrophy and right atrial enlargement; and (5) standard waveforms/complexes (three items): normal QRS complex, PR interval, and QT interval.

Each accurate response received one point. No supplementary weighting was implemented, even for life-threatening rhythms, to preserve scoring simplicity and compatibility across domains. The highest possible score was 15, and the participants were categorized as poor (score of 0-5), average (score of 6-10), and good (score of 11-15) in proficiency. Content validity was determined by a panel of three emergency medicine faculty members with competence in ECG teaching, who evaluated all items for clinical relevance and alignment with AHA guidelines.

Item difficulty was assessed by calculating the proportion of correct responses per item in the full sample (N=90). Items varied from effortless (e.g., normal sinus rhythm, 98.9% accuracy) to challenging (ventricular tachycardia, 27.8% accuracy), indicating appropriate variation across the knowledge spectrum. Item discrimination was assessed by item total correlations, revealing satisfactory values (>0.30) for the majority of questions, supporting construct validity. The questionnaire's internal consistency was assessed using Cronbach's alpha on a pilot sample of 10 respondents, resulting in a value of 0.804, indicating satisfactory reliability. For the main study sample of 90 responses collected, 88 cases (97.8%) were valid and included in the analysis, while two cases (2.2%) were excluded due to missing data, based on listwise deletion. To assess the internal consistency of the scale used in the study, Cronbach’s alpha was calculated for the 15 items. The analysis yielded a Cronbach’s alpha value of 0.676 for the sample (N=90), indicating an acceptable level of reliability.

The decision to allot uniform weight (one point) to each question reached consensus during the validation phase. While life-threatening arrhythmias such as ventricular fibrillation or ventricular tachycardia possess higher clinical urgency, assigning unequal weight would complicate scoring and interpretation. This selection method offers a simple, replicable metric for evaluating ECG knowledge suitable for baseline competency assessment.

Data collection procedure

The survey was accessible for two weeks through a secure online link. Each invited participant was only allowed one entry. Submissions were systematically documented in Google Sheets (Google, Mountain View, CA) and subsequently exported to Microsoft Excel (Microsoft, Redmond, WA) for analysis.

Data management and security

The study population was categorized according to established performance score ranges to facilitate clear groupings of ECG interpretation proficiency: scores of 0-5 denoted poor, 6-10 indicated average, and 11-15 signified good proficiency. The selected cutoffs enable descriptive analysis and educational implications while maintaining the continuous nature of the underlying data for inferential statistics.

Participant roles were assigned numerical codes to facilitate systematic subgroup analyses of different cohorts, including second-year students, third-year students, interns, EMT staff, and nursing staff. This stratification recognizes the variability in clinical experience and education among groups, which likely affects ECG interpretation performance.

The stratification approach, along with meticulous data curation and secure management, enhances the validity of the study findings and establishes a clear framework for educational recommendations suited to various learner groups.

Incomplete, duplicate, or ineligible responses were rigorously excluded to maintain data integrity. The remaining dataset included only fully completed questionnaires, ensuring high-quality and reliable analysis.

All collected data were managed with stringent confidentiality protocols. Data were anonymized and securely stored on password-protected institutional servers, with regular backups, in compliance with local regulations and institutional policies regarding research data security and participant privacy.

Statistical analysis

Statistical analysis was performed using IBM SPSS version 20.0 software (IBM Corp, Armonk, NY). Categorical variables are presented as frequencies and percentages, while numerical variables are expressed as mean±standard deviation or median with interquartile range, depending on data distribution. Normality of continuous variables was assessed using the Shapiro-Wilk test. Due to violations of normality in some groups, nonparametric tests were applied for group comparisons. Differences in total scores across more than two independent groups were assessed using the Kruskal-Wallis test. Where significant, post-hoc pairwise comparisons were conducted using Dunn’s test with Bonferroni correction to adjust for multiple testing. The strength and direction of association between total score and age were evaluated using Spearman’s rank correlation coefficient. A p-value <0.05 was considered statistically significant for all analyses.

## Results

The study comprised 90 volunteers, encompassing diverse educational and professional backgrounds in emergency and intensive care services at a tertiary care teaching hospital (Table 1). The sample included 21 second-year students (23.3%), 19 third-year students (21.1%), 19 interns (21.1%), and 31 staff members (34.5%), facilitating significant comparisons of ECG interpretation skills across various training levels and professional experiences.

**Table 1:** Distribution of Study Participants by Group Number and percentage of study participants categorized by educational year and professional role, including second-year students, third-year students, interns, and staff members (N=90).

Group	Number (n)	Percentage (%)
Second-year students	21	23.3
Third year students	19	21.1
Interns	19	21.1
Staff members	31	34.5
Total	90	100

Table 2 summarizes the descriptive statistics of overall ECG interpretation scores for each group. Second-year students recorded the lowest mean score of 9.57 (SD=2.34, 95% Cl: 8.51-10.63), while third-year students achieved the greatest mean score of 13.21 (SD=1.87, 95% Cl: 12.31-14.11). Interns (mean=12.26, SD=1.59, 95% Cl: 11.50-13.03) and staff members (mean=12.32, SD=1.92, 95% Cl: 11.62-13.03) achieved similar scores. Participants demonstrated excellent proficiency in identifying fundamental ECG rhythms. Participants exhibited exceptional accuracy in recognizing normal sinus rhythm, left ventricular hypertrophy, and normal QRS complexes, indicating a robust understanding of fundamental ECG patterns.

**Table 2:** . Descriptive Statistics of Total ECG Interpretation Scores by Participant Group The table presents summary statistics of total ECG interpretation scores (range, mean, standard deviation, and 95% confidence interval) for each participant group, illustrating differences in ECG proficiency by training level (N=90).

Group	n	Minimum	Maximum	Mean±SD	95% CI	
					Lower bound	Upper bound
Second-year students	21	6	15	9.57±2.336	8.51	10.63
Third-year students	19	8	15	13.21±1.873	12.31	14.11
Interns	19	8	15	12.26±1.593	11.50	13.03
Staff members	31	8	15	12.32±1.922	11.62	13.03
	N=90					

However, performance markedly declined in recognizing complex arrhythmias such as ventricular tachycardia and modest ST-T alterations, which are essential for detecting life-threatening conditions. The challenges were especially notable, indicating targeted educational interventions are needed to bridge these gaps.

Across all 15 ECG interpretation questions (Table 3), the overall correct response rate ranged from 27.8% to 98.9%. The highest accuracy was observed for Q12 (identification of normal sinus rhythm; 98.9%), Q14 (detection of left ventricular hypertrophy; 94.4%), and Q15 (recognition of normal QRS; 93.3%), reflecting strong mastery of fundamental ECG concepts. In contrast, Q10 (identification of ventricular tachycardia; 27.8%) and Q8 (recognition of subtle ST-T changes; 51.1%) were the most challenging, indicating difficulties in interpreting complex ECG abnormalities. The correct response rates for most of the other questions were over 70%, which suggests that the group as a whole had a good understanding of ECGs.

**Table 3:** Frequency and Proportion of Correct Responses for Each ECG Interpretation Question Overall counts and percentages of participants correctly answering each ECG interpretation question (Q1–Q15), highlighting areas of strong and weak performance in the full sample (N=90).

ECG	Points	Correct answer, n (%)
ECG 1	1	73 (81.1)
ECG 2	1	81 (90.0)
ECG 3	1	69 (76.7)
ECG 4	1	75 (83.3)
ECG 5	1	80 (88.9)
ECG 6	1	80 (88.9)
ECG 7	1	64 (71.1)
ECG 8	1	46 (51.1)
ECG 9	1	80 (88.9)
ECG 10	1	25 (27.8)
ECG 11	1	67 (74.4)
ECG 12	1	89 (98.9)
ECG 13	1	69 (76.7)
ECG 14	1	85 (94.4)
ECG 15	1	84 (93.3)
	Total score: 15	

Table 4 delineates the performance variation per group for each question. Third-year students, interns, and staff members attained above 85% accuracy for majority of the questions, although second-year students exhibited diminished proficiency on some difficult items, notably Q1 (47.6%), Q7 (28.6%), Q8 (33.3%), Q10 (19.0%), and Q11 (30.0%). Questions 7, 8, and 10 posed difficulties for all groups, but questions 12, 14, and 15 were consistently answered correctly by over 90% of participants in each group. The trends indicate that focused educational interventions are essential for developing sophisticated interpretation skills during initial training phases.

**Table 4:** : Percentage of Correct Responses for each ECG Question by participant by group Detailed percentages of correct responses for each ECG question broken down by participant groups, showing performance variation across training levels.  Percentage score marked with an asterisk (*) represents questions that were answered with peak accuracy among different participant groups

Question	Second-Year students (%)	Third-Year students (%)	Interns (%)	Staff members (%)	Overall (%)
Q1	47.6	89.5	94.7	90.3	81.1
Q2	85.7	94.7	94.7	87.1	90.0
Q3	85.7	89.5	68.4	67.7	76.7
Q4	85.7	94.7	84.2	74.2	83.3
Q5	71.4	94.7	100.0	90.3	88.9
Q6	71.4	94.7	94.7	93.5	88.9
Q7	28.6	84.2	73.7	90.3	71.1
Q8	33.3	68.4	31.6	64.5	51.1
Q9	85.7	84.2	78.9	100.0	88.9
Q10	19.0	63.2	42.1	3.2	27.8
Q11	30.0	89.5	89.5	87.1	75.3
Q12	95.2	100.0	100.0	100.0	98.9*
Q13	47.6	84.2	84.2	87.1	76.7
Q14	90.0	94.7	94.7	100.0	95.5*
Q15	85.7	94.7	94.7	96.8	93.3*

**Figure 1:**
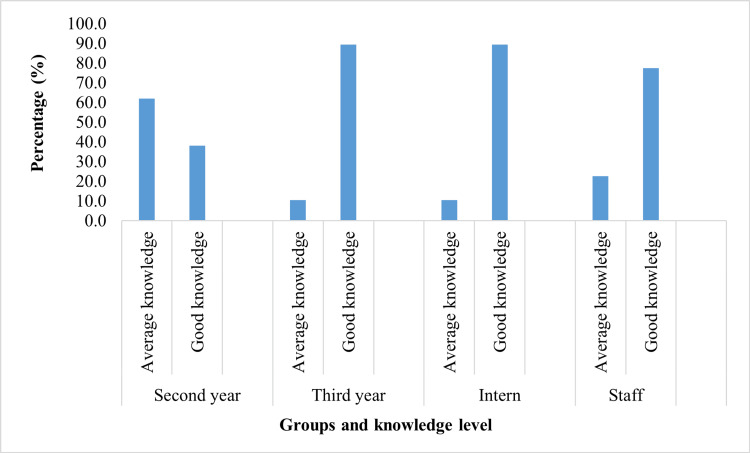
Proportion of Average and Good Knowledge Levels Among Various Groups Knowledge levels of ECG interpretation were compared across second-year and third-year students, interns, and staff members.

The bar chart (Figure 1) illustrates the proportion of paramedics categorized as having average and good knowledge of ECG interpretation. The X-axis represents the group and knowledge level, and the Y-axis indicates the associated percentage. The chart indicates that third-year students, interns, and staff members exhibit a significantly higher proportion of proficient knowledge, while a majority of second-year students possess average knowledge.

The group-wise distribution (Table 5) indicated that the majority of third-year students (89.5%), interns (89.5%), and staff members (77.4%) were classified inside the good knowledge category, in contrast to merely 38.1% of second-year students.

**Table 5:** Knowledge Level Distribution by Group Frequencies and percentages of participants with average or proficient ECG interpretation knowledge, compared across various groups

Year	Knowledge group	Frequency	Percentage(%)
Second-year students	Average knowledge	13	61.9
	Good knowledge	8	38.1
Third-year students	Average knowledge	2	10.5
	Good knowledge	17	89.5
Interns	Average knowledge	2	10.5
	Good knowledge	17	89.5
Staff members	Average knowledge	7	22.6
	Good knowledge	24	77.4

Upon evaluating all participants collectively (Table 6), 73.3% (n=66) demonstrated good knowledge, whereas 26.7% (n=24) exhibited average knowledge, signifying a robust proficiency in ECG interpretation.

**Table 6:** Overall Knowledge Profile Among the Groups Summary of the collective knowledge distribution among all participants, reflecting the ratio of those with average versus proficient ECG interpretation expertise (N=90).

Knowledge group	Frequency	Percentage
Average Knowledge	24	26.7
Good knowledge	66	73.3
Total	90	100

A Spearman's rank-order correlation was performed to analyze the association between age and total ECG interpretation score. The connection was weakly positive (ρ=0.183, p=0.083, N=90) and did not achieve statistical significance, indicating that age was not a determinant of ECG proficiency in this group.

**Figure 2:**
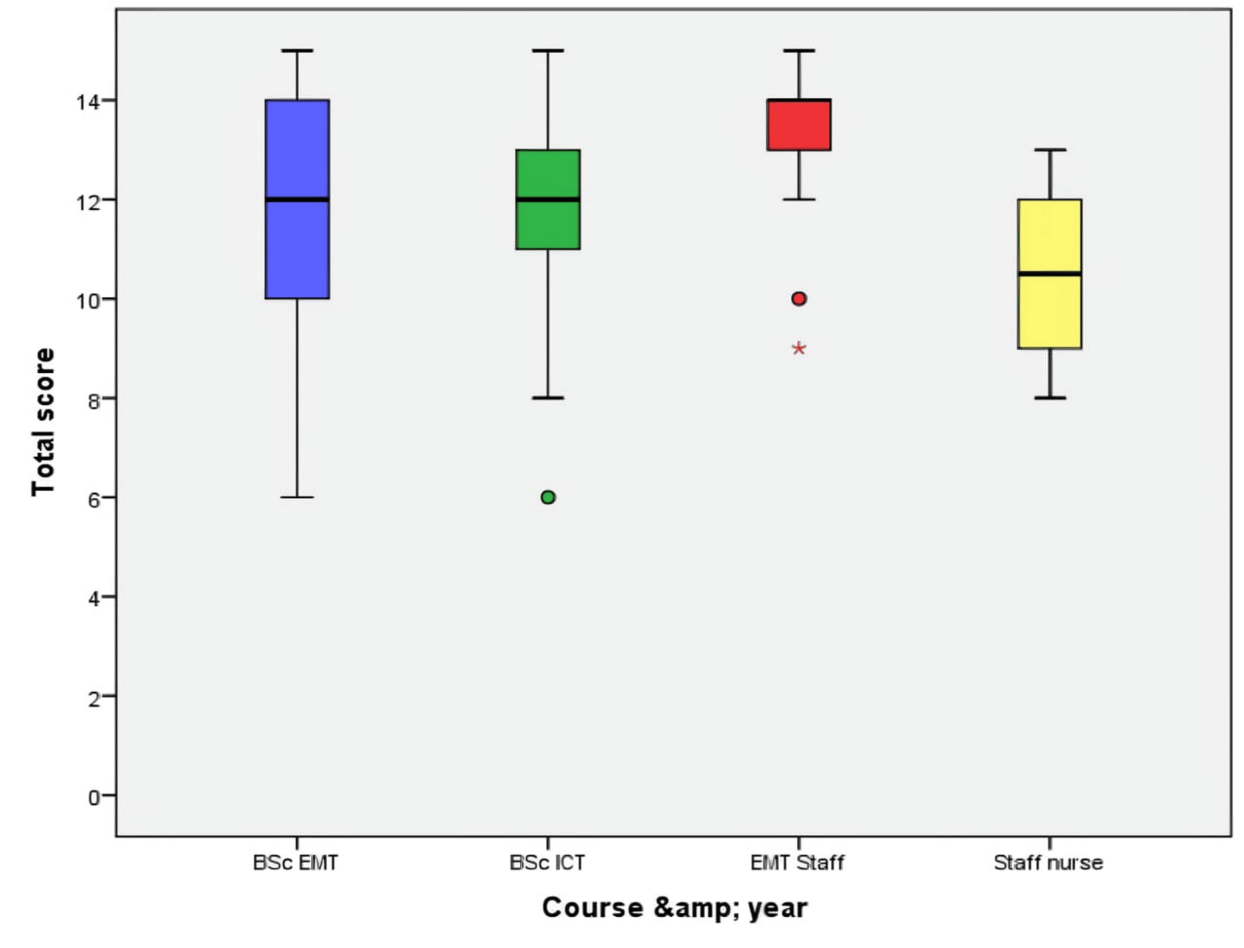
Boxplot Depicting ECG Scores of Participants Classified by Department and Role A boxplot illustrates the distribution of total ECG scores across different groups. The central line represents the median, the box represents the interquartile range, and whiskers indicate the main range of scores. As an example, mild outliers are shown as small circles, and extreme outliers as asterisks (*), representing values that are considerably different from the rest of the data. Median ECG scores were lowest among staff nurses.

**Figure 3:**
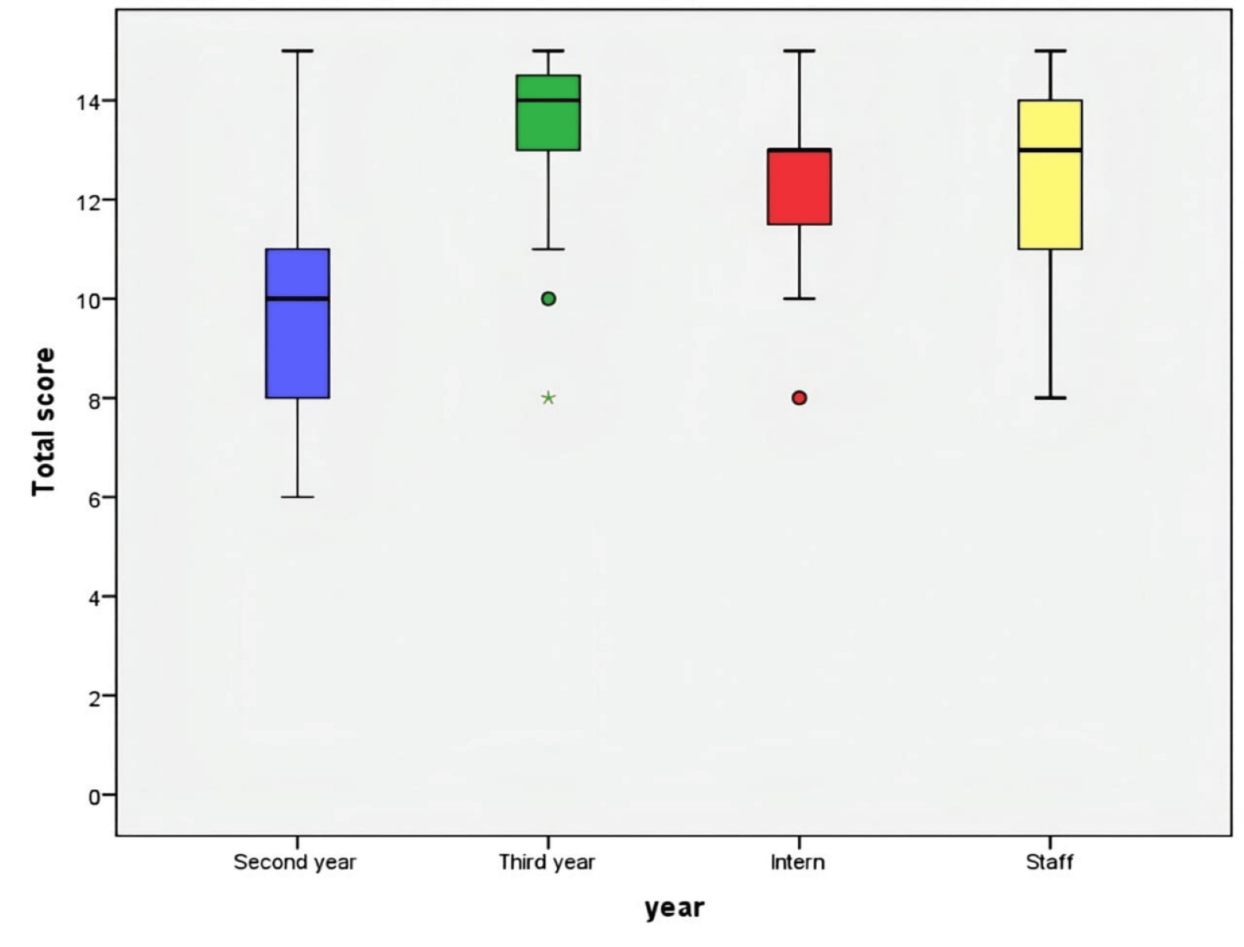
Boxplot Depicting Total Score by Groups as per Academic Year and Staff Status The boxplot depicts score percentiles by group, highlighting differences in median and variability among staff, interns, and student cohorts. Medians, interquartile ranges, and overall score distributions are shown for each cohort. Mild and extreme outliers are marked by circles and asterisks (*) respectively. The second-year group had the lowest median performance, while the third-year group showed the highest average scores.

The Kruskal-Wallis test revealed a statistically significant difference in overall ECG interpretation scores across the four groups (χ²(3)=11.954, p=0.008), which indicates how the training level and professional roles significantly affected competency. Figures 2, 3 illustrate the study of percentile distribution and the visual examination of score dispersion.

The boxplot (Figure 2) and Table 7 demonstrate that EMT employees attained the highest medians and the narrowest interquartile ranges, signifying sustained high performance. Staff nurses exhibited the lowest medians and the greatest variability, indicating both inferior and more fluctuating results. These visual trends correspond with the data findings, validating superior performance and consistency at elevated levels of training and experience.

**Table 7:** Distribution of Participants by ECG interpretation Skill Level Across Different Training Groups Statistical differences among groups were analyzed using the non-parametric Kruskal-Wallis test due to non-normal data distribution. EMT: Emergency medical technology; ICT: Intensive care technology; EMT staff: emergency medical technician staff; CI: confidence interval.

Variable	Group	n	95% CI		Median(Q_1_, Q_2)_	χ²	df	ε²	p-value
			Lower bound	Upper bound					
Total score	BSc EMT	37	10.71	12.48	12 (10, 14)	11.954	3	0.134	0.008
	BSc ICT	22	10.63	12.65	12 (10.75, 13)				
	EMT staff	21	12.49	13.79	14 (13, 14)				
	Staff nurse	10	9.37	11.83	10.50 (9, 12.25)				

The Shapiro-Wilk test was used to check for normality by looking at whether the total ECG interpretation scores in each group followed a normal distribution. The results indicated that the EMT staff group (p<0.001) and the BSc EMT students (p=0.022) significantly deviated from normality, while the BSc ICT students (p=0.074) and staff nurses (p=0.591) did not demonstrate statistically significant departures from normality. Given these mixed results and the violation of normality assumptions in key groups, a non-parametric approach was deemed appropriate to compare scores across groups.

A Kruskal-Wallis test was conducted to compare total ECG interpretation scores among four groups representing different courses and roles: BSc EMT students (n=37), BSc ICT students (n=22), EMT staff (n=21), and staff nurses (n=10). Descriptive statistics revealed median scores of 12 (interquartile range (IQR): 10-14) for BSc EMT, 12 (IQR: 10.75-13) for BSc ICT, 14 (IQR: 13-14) for EMT staff, and 10.5 (IQR: 9-12.25) for staff nurses, indicating a trend of increasing scores from nursing staff through students to EMT staff.

The Kruskal-Wallis test showed a statistically significant difference in total scores among the groups (χ²(3)=11.954, p=0.008), with an epsilon squared effect size of 0.134, which corresponds to a moderate effect size. This suggests that group membership explains approximately 13.4% of the variance in ECG interpretation scores. Table 8 presents the pairwise comparison of ECG interpretation scores among various participant groups involved in the study using Dunn’s post-hoc test with Bonferroni adjustment. The analysis unveiled that the EMT staff scored significantly higher in contrast to the staff nurses (adjusted p=0.026), emphasizing prevalent ECG interpretation skills among EMT professionals.

**Table 8:** Pairwise Comparisons of ECG Interpretation Scores Among Participant Groups Post-hoc comparisons using Dunn’s test with Bonferroni adjustment revealed that EMT staff scored significantly higher than staff nurses (adjusted p=0.026). Differences between EMT staff and BSc ICT students (adjusted p=0.112) and between EMT staff and BSc EMT students (adjusted p=0.321) were not statistically significant after correction for multiple comparisons. No other pairwise comparisons reached statistical significance. EMT: Emergency medical technology; ICT: Intensive care technology; EMT staff: emergency medical technician staff.

Pairwise Group	Test statistic	p-value	Adj.p-value
Staff nurse - BSc EMT	1.823	0.177	1.000
Staff nurse - BSc ICT	2.327	0.127	0.763
Staff nurse - EMT Staff	8.119	0.004	0.026
BSc EMT - BSc ICT	0.001	0.971	1.000
BSc EMT - EMT Staff	3.728	0.053	0.321
BSc ICT - EMT Staff	5.532	0.019	0.112

A Kruskal-Wallis test was used to compare total ECG interpretation scores across four groups based on academic progression: second-year students (n=21), third-year students (n=19), interns (n=19), and staff members (n=31). Descriptive statistics indicated a distinct upward trajectory in performance corresponding to academic level. Median scores were 10 (IQR: 7.5-11) for second-year students, 14 (IQR: 13-15) for third-year students, 13 (IQR: 11-13) for interns, and 13 (IQR: 11-14) for staff members.

The Kruskal-Wallis test revealed a statistically significant difference in total scores across the groups (χ²(3)=26.024, p<0.001), with an epsilon squared (ε²) effect size of 0.292, indicating a large effect. This suggests that approximately 29.2% of the variance in ECG interpretation scores can be attributed to the participants' academic level or role (Table 9).

**Table 9:** Distribution of Participants by Academic Year and Staff Status Statistical differences among groups were analyzed using the non-parametric Kruskal-Wallis test due to non-normal data distribution. CI: confidence interval.

Variable	Group	n	95% CI		Median(Q_1_, Q_2)_	χ²	df	ε²	p-value
			Lower bound	Upper bound					
Total score	Second-year students	21	8.51	10.63	10 (7.50, 11)	26.024	3	0.292	<0.001
	Third-year students	19	12.31	14.11	14 (13, 15)				
	Interns	19	11.50	13.03	13 (11, 13)				
	Staff members	31	11.62	13.03	13 (11, 14)				

The examination of overall scores across groups categorized by academic year and staff status indicated a statistically significant difference (p<0.001). Among them, second-year students recorded the lowest median score of 10.0 (IQR: 7.5-11.0), whereas the highest scores were observed in third-year students, with a median of 14.0 (IQR: 13.0-15.0). Both interns and staff achieved nearly similar outcomes, each with a median score of 13.0; however, their interquartile ranges slightly differed (interns: 11.0-13.0; staff members: 11.0-14.0). Overall, the results highlight a strong link between academic standing and performance, with third-year students scoring the best and second-year students lagging behind.

The Shapiro-Wilk test was used to examine for normality in the distribution of total ECG interpretation scores for each academic level. The results indicated that the third-year group (p=0.004) and the staff group (p=0.001) showed significant departures from normality, while the second-year (p=0.478) and intern (p=0.107) groups did not. Due to the non-normal distribution across several groups, a non-parametric statistical method was suitable for group comparisons. Table 10 depicts group-wise comparison analysis of ECG interpretation scores with respect to different academic levels and staff groups using Dunn’s post-hoc test with Bonferroni adjustment. The analysis rendered marked improvement in ECG interpretation proficiency proportional to increased academic progression exposure. The second-year students scored significantly less than interns, staff and Third-year students. (adjusted p-value <0.001) for all comparisons.

**Table 10:** Pairwise Comparisons of ECG Interpretation Scores between Academic Year and Staff Status Post-hoc pairwise comparisons using Dunn’s test with Bonferroni correction revealed that second-year students scored significantly lower than interns (adjusted p=0.001), staff (adjusted p<0.001), and third-year students (adjusted p<0.001). No statistically significant differences were observed among the more senior groups: interns vs. staff (adjusted p=0.793), interns vs. third-year students (adjusted p=0.100), and staff vs. third-year students (adjusted p=1.000).

Pairwise Group	Test statistic	p-value	Adj.p-value
Second-year students - Interns	14.401	<0.001	0.001
Second-year students - staff members	16.113	<0.001	<0.001
Second-year students - third-year students	19.558	<0.001	<0.001
Interns - staff members	2.266	0.132	0.793
Interns - third-sear students	5.729	0.017	0.100
Staff members - third-year students	1.422	0.233	1.000

The results indicate a distinct trend of enhanced ECG interpretation ability correlated with higher academic levels and greater professional experience. Despite the robust foundational knowledge of ECG among the cohort, the findings underscore the necessity for focused educational initiatives to enhance advanced interpretative skills, especially among students in their initial years.

## Discussion

This study revealed a notable advancement in ECG interpretation skills throughout various academic years and professional tiers in emergency and intensive care environments. Among the participant groups, third-year students, interns, and staff members demonstrated marked superior accuracy compared to second-year students, thereby affirming that ECG proficiency progressively enhances with systematic instruction and clinical experience. This study corroborates previous research by Coll-Badell et al. [12] and Pitel et al. [13], which documented significant skill enhancement associated with curricular and practical experience, emphasizing the necessity of integrated theoretical and experiential training.

Our findings indicated that fundamental ECG concepts such as normal sinus rhythm, left ventricular hypertrophy, and normal QRS complexes were accurately identified by over 90% of participants, aligning with observations by Kashou et al. [14], who reported early proficiency in these essentials among medical and paramedical students. Participants encountered significant difficulties with advanced ECG abnormalities, including ventricular tachycardia (Q10; 27.8% correct) and subtle ST-T changes (Q8; 51.1%), reflecting challenges reported globally by Oh et al. [4] and Kopeć et al. [5], which highlight ongoing educational deficiencies in the recognition of complex arrhythmia [9].

Notably, merely 3.2% of personnel accurately recognized the ECG rhythm indicative of ventricular tachycardia (Q10), signifying the lowest precision among all evaluated items. These findings hold substantial clinical significance, as the rapid recognition of ventricular tachycardia is essential for timely defibrillation or antiarrhythmic therapy, key determinants of survival during cardiac arrest situations. The low identification rate indicates a significant learning deficiency among emergency department staff in distinguishing wide-QRS tachyarrhythmias. However, this result may also be influenced by item ambiguity or possible misclassification within the assessment tool. From an educational perspective, these findings highlight the pressing necessity to improve instruction in distinguishing shockable rhythms via case-based learning, high-fidelity simulation, and supervised rhythm workshops. Given that all participants were professionals or trainees in emergency medicine departments, this observation holds obvious clinical significance: accurately identifying ventricular tachycardia can determine the difference between delayed and prompt resuscitation success.

Second-year students exhibited much poorer accuracy on advanced ECG questions, reflecting findings from research by Kashou et al. [14] and Abdalla et al. [15], which indicate serious early training inadequacies that could jeopardize clinical preparedness if not remedied through an enhanced curricular emphasis. This underscores the pressing necessity to develop intricate ECG interpretation abilities early and to reinforce them through repeated exposure to clinical contexts [4,5]. Interventional instructional approaches, such as workshops and supervised practice sessions, substantially enhance ECG diagnostic accuracy among interns and postgraduate students. These findings support the institutional deployment of regular training sessions overseen by cardiology specialists to sustain and improve interpretation skills [16].

Based on Chudgar et al.'s [17] reports, no statistically significant correlation was found between age and ECG proficiency. In Ahmad et al. [18], the results indicate that chronological age does not predict interpretive ability, but rather deliberate practice and structured education do, reflecting expert skill acquisition and deliberate practice theory [19,20].

The Kruskal-Wallis test indicated significant differences in scores among participant groups (χ²(3)=11.954, p=0.008), corroborating Kaye et al.'s [21] advocacy for a tiered, competency-based ECG curriculum designed for learners' developmental phases. To enhance learners' readiness for real-world scenarios, Q7, Q8, and Q10 provide empirical targets for focused educational improvements [22].

The findings are consistent with constructivist and experiential learning theories, highlighting the importance of active involvement and iterative feedback in the acquisition of difficult abilities. Innovative pedagogies, including simulation-based training, flipped classrooms, case-based learning, and gamification, have shown enhanced effectiveness in ECG teaching by fostering engagement and facilitating repeated practice essential for mastering, as evidenced by systematic reviews and meta-analyses [22-24].

The greater ECG proficiency of interns and staff clinically illustrates the importance of supervised clinical practice and expert supervision in converting theoretical knowledge into diagnostic accuracy and confidence, as noted by Saravi et al. [25]. Incorporating systematic ECG interpretation exercises with mentorship during clinical rotations enhances diagnostic skills essential for patient outcomes.

The study's methodological strengths encompass a diverse participant sample spanning academic and professional tiers, thorough group-level analysis identifying knowledge deficiencies, and validated instruments demonstrating high reliability (Cronbach’s alpha=0.804). Nonetheless, its single-center, cross-sectional design restricts generalizability, and binary scoring may conceal subtleties of partial knowledge or misconceptions. Future longitudinal and multicenter investigations employing refined evaluation methodologies are necessary for enhanced understanding of ECG skill improvement.

Summary of findings and future directions

This study evaluated ECG interpretation proficiency among 90 participants, comprising paramedic students, interns, staff members, and ER nurses. The overall proficiency was elevated, with 73.3% exhibiting strong knowledge and 26.7% displaying average knowledge, while no individuals fell into the poor category. Performance is enhanced with increased training and clinical experience, as third-year students (mean score 13.21), interns (12.26), and staff (12.32) are considerably above second-year students (9.57; p<0.001). Fundamental rhythms, including normal sinus rhythm, normal QRS complexes, and left ventricular hypertrophy, were identified with over 90% accuracy, indicating robust foundational proficiency. Nonetheless, advanced arrhythmias presented significant difficulty, with ventricular tachycardia accurately recognized by merely 27.8% of the participants and modest ST-T alterations by 51.1% participants. Emergency room nurses and second-year students generally achieved poorer scores, especially in recognizing intricate rhythms, indicating inadequate readiness for real-time cardiac problems. These findings highlight that while paramedic students, interns, EMT personnel, and emergency room nurses generally excel at recognizing fundamental patterns, substantial shortcomings remain in detecting life-threatening rhythms, which directly impact the timeliness of cardiac arrest recognition, the initiation of CPR, and adherence to ACLS protocols.

The proficiency scores identified in this study have significant consequences for clinical practice. Elevated proficiency in ECG interpretation among advanced students, interns, and EMT personnel signifies preparedness to identify and swiftly address life-threatening arrhythmias, essential for minimizing delays in emergency care. The reduced accuracy observed in second-year students and ER nurses indicates that, in actual clinical situations, these cohorts may be inadequately prepared to recognize small but vital ECG alterations, which could result in postponed or erroneous interventions during cardiac emergencies. Establishing a baseline standard of ECG interpretation skill at all levels is crucial for prompt cardiac arrest identification and the efficient implementation of advanced life support protocols.

To overcome these deficiencies, future initiatives should concentrate on bridging the gaps in advanced ECG interpretation, particularly in the recognition of ventricular tachycardia and subtle ischemic changes, which are essential for the timely recognition of cardiac arrest rhythms. The early integration of structured case discussions, high-fidelity mock codes, and simulation-based training into the curriculum can strengthen rhythm interpretation skills for providers encountering real-life emergencies. Alongside information gain, cultivating self-confidence is essential for proficient ECG interpretation in high-pressure clinical situations. Educational interventions that integrate theoretical knowledge with simulation and case-based learning augment both competence and clinician confidence, ultimately enhancing diagnostic decision-making and patient outcomes [16]. Blending ECG training with practical resuscitation exercises will reinforce both diagnostic accuracy and the initiation of high-quality chest compression. 

To rectify detected inadequacies, it is advisable to establish regular, level-specific ECG interpretation seminars, particularly aimed at early-stage students and emergency room nurses. Simulation-based instruction, utilizing high-fidelity mock codes and case scenario rehearsals, can significantly enhance diagnosis speed and precision, particularly for complex arrhythmias. Requiring regular formative assessments and using digital learning modules on complex ECG subjects will promote continuous skill enhancement. Incorporating ECG interpretation competency criteria into clinical rotation assessments and staff performance evaluations will ensure the maintenance of elevated skill levels throughout all provider categories.

Strengths and limitations

The key strength of this study lies in its inclusion of a heterogeneous spectrum of participants comprising paramedic students, interns, certified emergency medical technicians (EMT staff), and emergency room nursing personnel, all actively engaged in emergency medicine departments. This collaborative clinical setting underscores the essential necessity of ECG interpretation proficiency among frontline practitioners engaged in life-saving interventions. The assessment tool was prepared in accordance with the 2020 American Heart Association guidelines, ensuring clinical relevance and adherence to contemporary best practices. The tool exhibited strong internal consistency during the pilot phase (Cronbach’s alpha=0.804) and adequate reliability in the complete sample (Cronbach’s alpha=0.676), thereby affirming the general robustness of the measurement. The application of suitable non-parametric statistical techniques enhanced the robustness of the results.

Nonetheless, certain limitations must be acknowledged. The single-center, convenience sampling methodology may introduce selection bias, thereby limiting the generalizability of the study's findings to broader populations. Despite extensive participation from invited individuals, self-selection bias may still exist, potentially distorting outcomes in favor of those with more motivation or initial competence. The study did not document or control for prior ACLS or ECG interpretation training among participants, which could confound differences observed across groups. Moreover, the online, unproctored format of questionnaire administration introduces the potential for participants to utilize other resources, which may artificially enhance knowledge scores and exaggerate true competence. The binary scoring system employed, while straightforward, may underrepresent partial knowledge and nuances in the interpretation of complex ECG rhythms. The limited sample size constrains comprehensive subgroup analyses and diminishes the ability to identify smaller yet clinically significant effects. Finally, as a cross-sectional study, it cannot assess longitudinal learning retention or the translation of ECG interpretation skills to clinical practice, suggesting the need for future multicenter, longitudinal studies with proctored assessments and real-time performance evaluations to fully capture skill acquisition and clinical impact.

## Conclusions

This study confirms that the majority of paramedics and emergency nursing personnel exhibit proficient skills in identifying essential ECG rhythms, indicating effective foundational training and knowledge retention. Nonetheless, significant gaps persist in the accurate recognition of advanced arrhythmias, such as ventricular tachycardia and modest ST-T segment alterations, especially among novice trainees and ER nurses. Given that the timely recognition of these arrhythmias is critical to the initiation of ACLS protocols and improved patient outcomes, these deficiencies pose a substantial clinical issue.

To address these gaps, it is essential to provide organized educational interventions that include continuous, tiered training, comprising a minimum of 20 hours annually focused on ECG interpretation practice. This should underscore simulation-based scenarios focused on high-risk arrhythmias, regular competency evaluations, and the integration of these competencies into clinical rotation assessments and staff performance reviews.

Targeted training programs will improve interpretative accuracy and enhance clinicians' confidence in emergency decision-making. Ultimately, these measures aim to improve the timeliness and quality of resuscitative efforts, thereby reducing morbidity and mortality in cardiac emergencies.

## Figures and Tables

**Figure 4:**
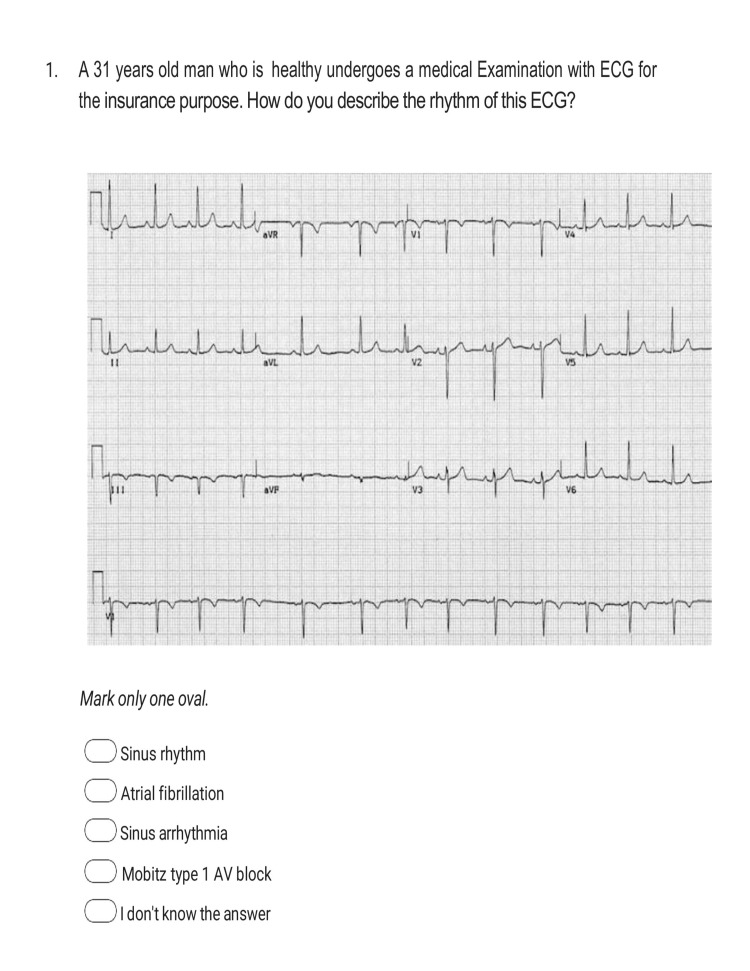
Question number 1 Questionnaire administered to study participants. Questionnaire page no 1.

**Figure 5:**
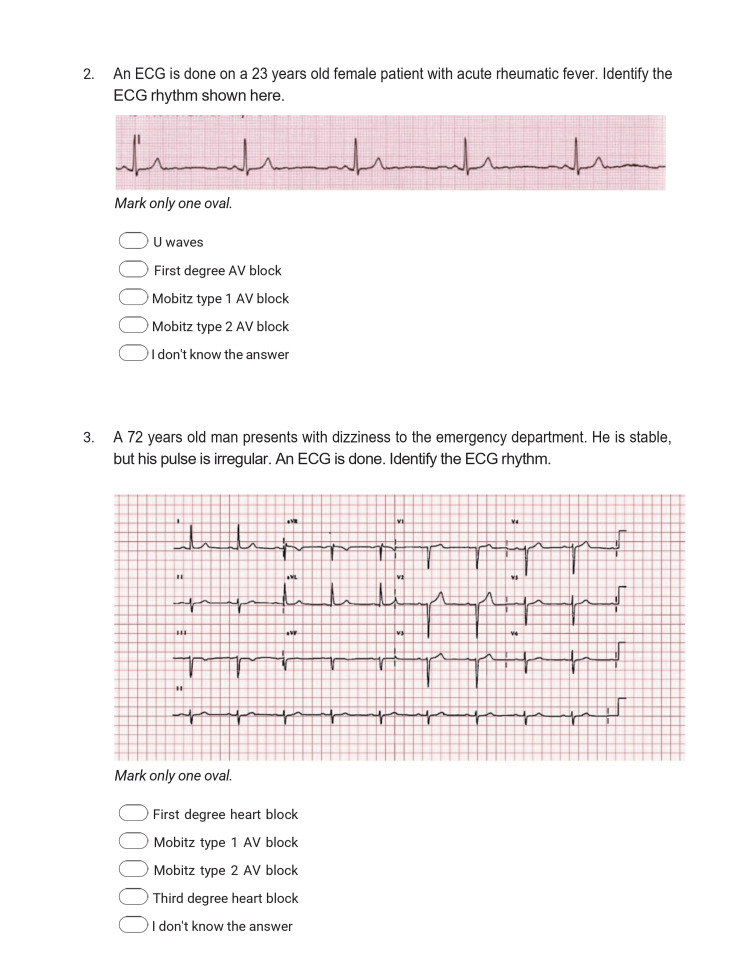
Question numbers 2 and 3 Questionnaire page no. 2.

**Figure 6:**
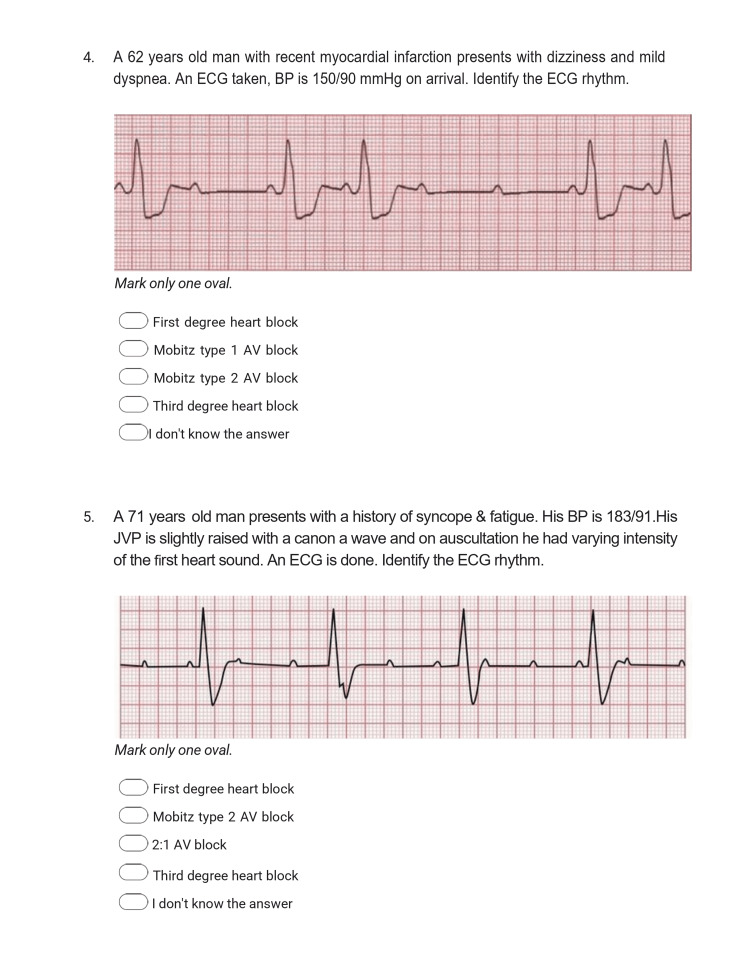
Question numbers 4 and 5 Questionnaire page no. 3.

**Figure 7:**
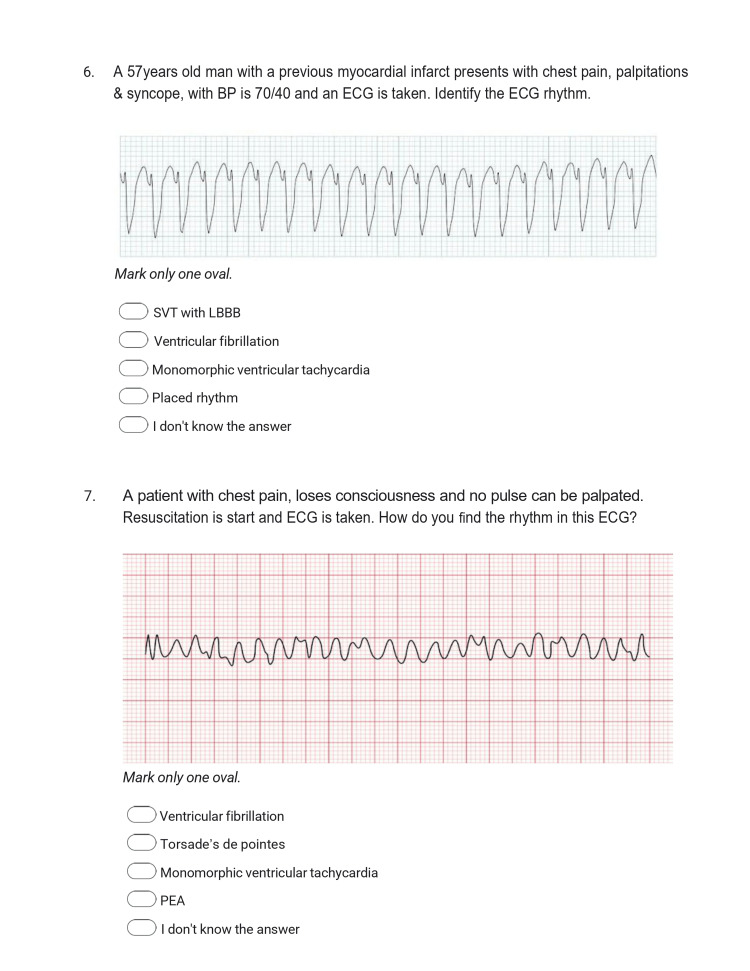
Question numbers 6 and 7 Questionnaire page no. 4.

**Figure 8:**
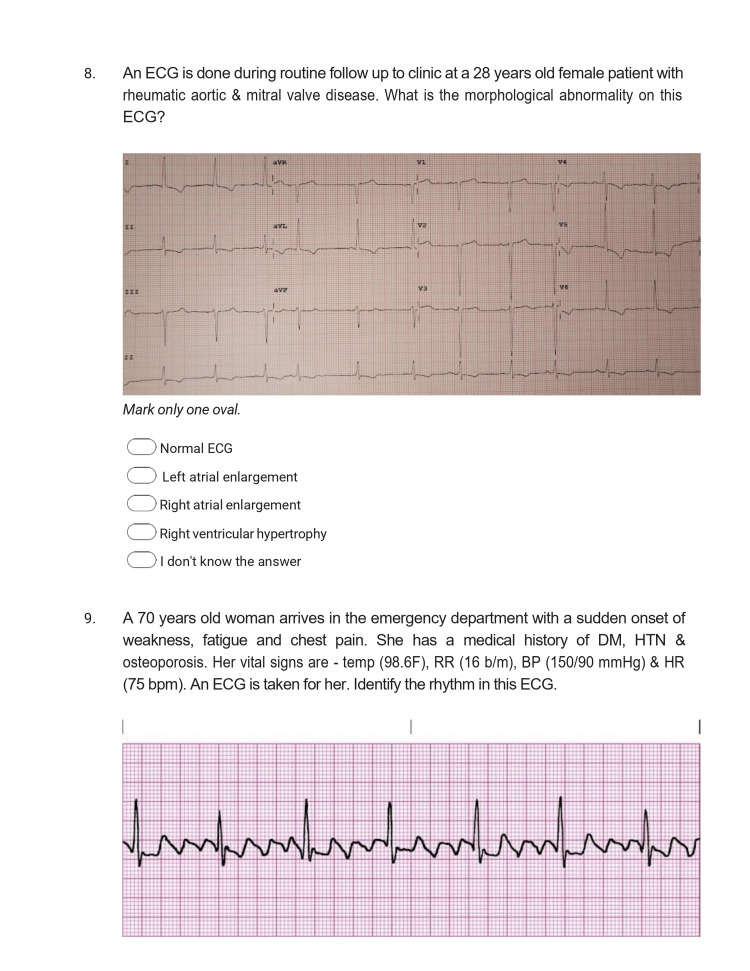
Question numbers 8 and 9 Questionnaire page no. 5.

**Figure 9:**
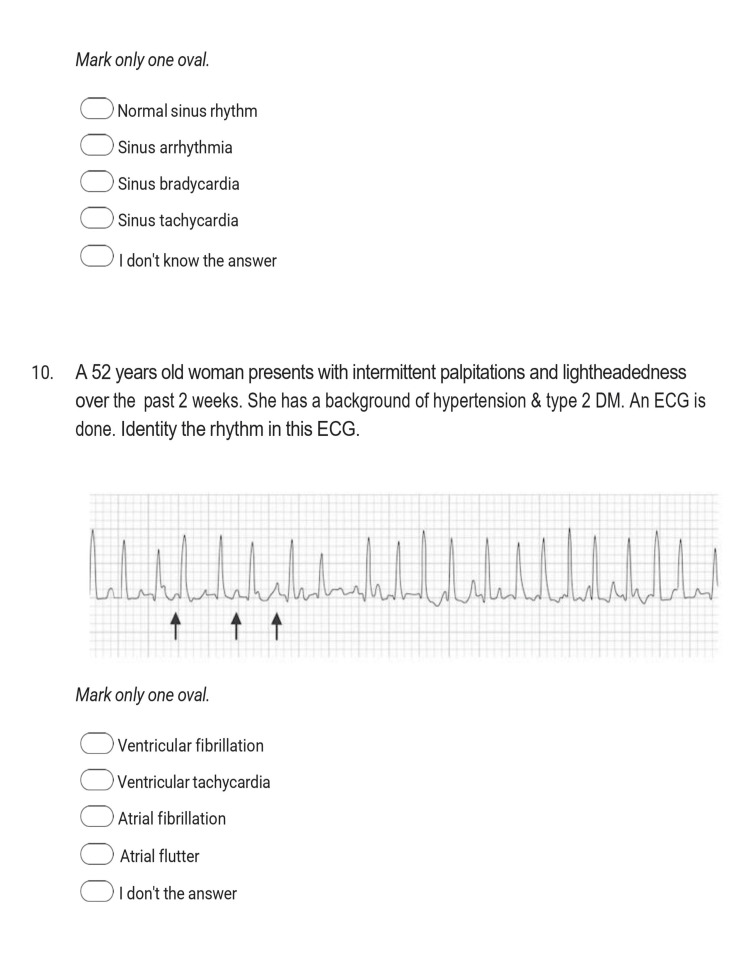
Question number 10 Questionnaire page no. 6.

**Figure 10:**
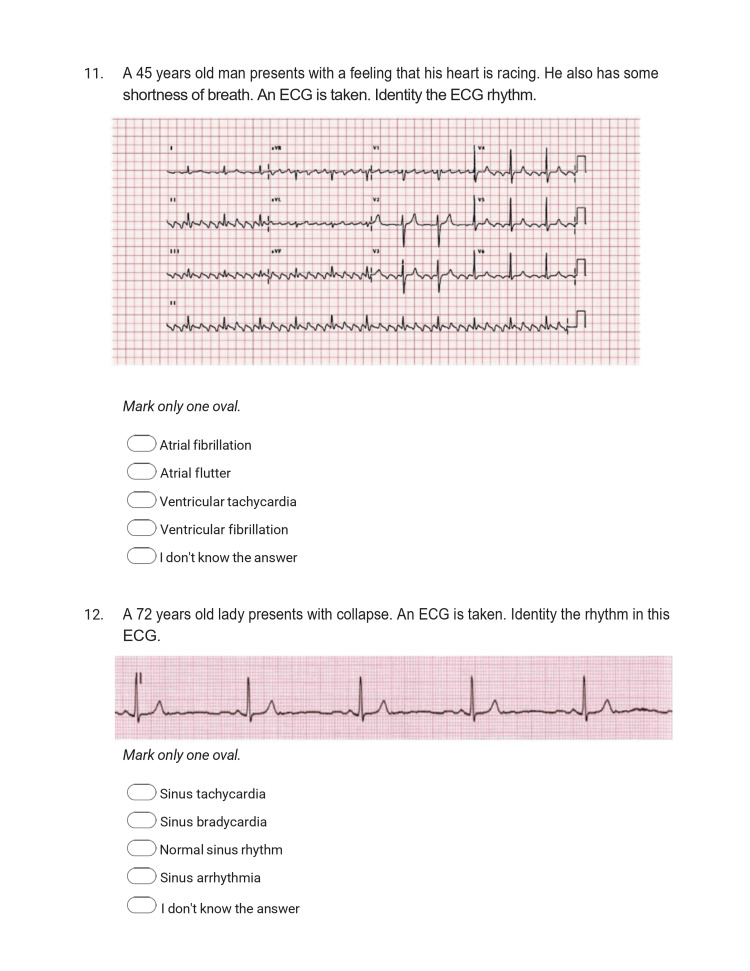
Question numbers 11 and 12 Questionnaire page no. 7.

**Figure 11:**
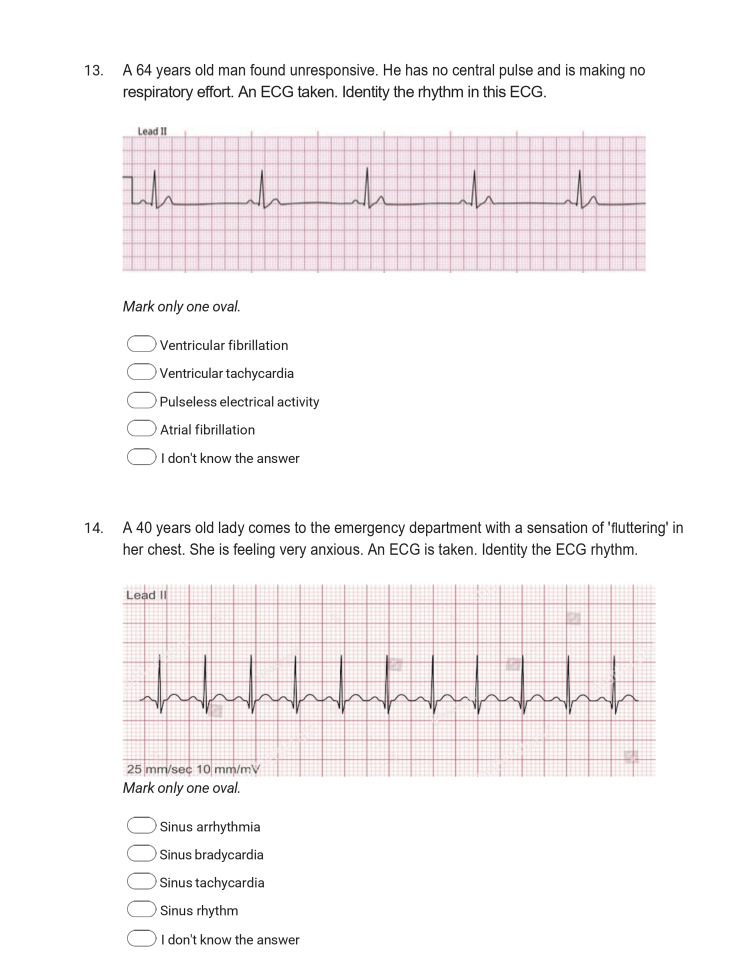
Question numbers 13 and 14 Questionnaire page no. 8.

**Figure 12:**
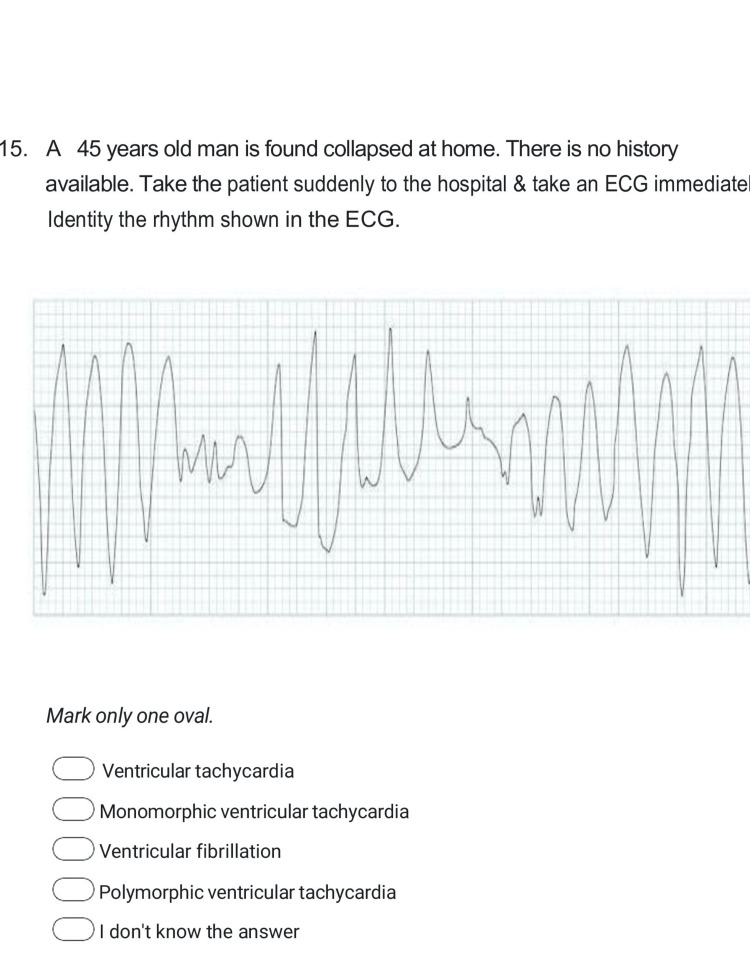
Question number 15 Questionnaire page no. 9.
